# Bowel preparation for colonoscopy may decrease the levels of testosterone in Korean men

**DOI:** 10.1038/s41598-019-43598-5

**Published:** 2019-05-08

**Authors:** Soo-Hyun Lee, Seung Geon Park, Moon-Jong Kim, Hyejin Chun, Doo-Yeoun Cho, Doohee Hong, Young-Sang Kim

**Affiliations:** 1 Department of Family Medicine, CHA Bundang Medical Center, CHA University, Seongnam, 13496 Republic of Korea; 2 Department of Clinical Pharmacology and Therapeutics, CHA Bundang Medical Center, CHA University, Seongnam, 13496 Republic of Korea; 3 Suwon Central Hospital, Suwon, 16391 Republic of Korea

**Keywords:** Hypogonadism, Risk factors

## Abstract

Although colonoscopy is commonly conducted for medical check-ups in Korea, investigations for the influence of bowel preparation on various health conditions are insufficient. This cross-sectional study investigated whether bowel preparation has an influence on serum levels of testosterone. A total of 1114 men were divided into the bowel preparation group and control groups. The median total and free testosterone levels were significantly lower in the bowel preparation group (14.89 and 0.26 nmol/L, respectively) than in the control groups (15.72 and 0.28 nmol/L, respectively). The level of total testosterone significantly increased with age in the bowel preparation group (r = 0.103). The differences in the levels of total and free testosterone between the 2 groups were more prominent in younger men than in older men. In multivariate regression models, bowel preparation was independently associated with the levels of total and free testosterone. In these models, the interaction between age and bowel preparation was significant for the levels of total and free testosterone. In conclusion, bowel preparation may independently decrease the serum levels of total and free testosterone. The decline in testosterone was more evident in younger men than in older men.

## Introduction

Androgens play an important role in the male reproductive system, sexual functions, body compositions, and behaviours^1,2^. Low levels of circulating androgens may cause weakness, fatigue, anaemia, hair loss, decreased bone density and lean body mass, increased fat mass, and sexual dysfunction^3–5^. Hence, men with these symptoms may be suspected to have androgen deficiency. Men with the above mentioned clinical symptoms and deficiency of circulating androgen are defined as having hypogonadism^6,7^. In general, androgen deficiency is assessed by measuring serum total testosterone (TT) concentration^6,7^. In men who clinically manifest hypogonadism but do not present low levels of serum TT, the repeated measurement of TT and additional measurements of sex hormone-binding globulin (SHBG) or free testosterone (FT) are recommended^7^.

Serum testosterone levels are influenced by a variety of medical conditions and medications^8,9^. Previous studies have shown that testosterone levels may be reduced in certain stress conditions such as critical illness including acute myocardial infarction, surgical stress, and brain injury^10^. Physiological stress may modify testis functionality and disrupt the hypothalamic-pituitary-gonadal (HPG) axis^11–15^.

Colonoscopy is one of the useful methods for detection of colorectal cancers^16^. As colorectal polyps can be removed during the procedure, colonoscopy is commonly conducted for medical check-ups in Korea^17^. However, colonoscopy may result in adverse events such as abdominal discomfort or pain, perianal irritation, bleeding, and a tear in the colon wall^18–20^. In addition, adequate bowel preparation is essential for clear visualization of mucosal lesion^21,22^. Bowel preparation can also cause adverse events such as dehydration and electrolyte imbalance^23–26^.

Under the recent health screening procedure in Korea, drawing blood and colonoscopy are usually performed serially. Previous researches investigated the influence of such adverse effects, as dehydration and electrolyte imbalances on the levels of testosterone^27–29^, therefore we suspected that bowel preparation may affect the concentrations of serum testosterone. This study aimed to examine whether the physiological conditions induced by bowel preparation for colonoscopy have an influence on serum concentrations of TT and FT. We also investigated which factors other than bowel preparation may determine a difference in serum TT and FT concentration.

## Results

The general characteristics of the participants are presented in Table 1. The median age was 51 years in both groups. The median TT levels were 14.89 and 15.72 ng/mL in the bowel preparation and control groups (P < 0.001). SHBG levels were not different between the 2 groups. FT levels were significantly different between the 2 groups (0.256 and 0.279 ng/mL in bowel preparation and control group, respectively). The bowel preparation group had a significantly higher phosphorus level (2.10 mmol/L) than the control group (1.07 mmol/L). Levels of calcium, sodium, and potassium were significantly different between the 2 groups.

**Table 1:** General characteristics of the study population.

	Bowel preparation (−)	Bowel preparation (+)	P
	N = 386	N = 728	
Age	51.0 (45.0–61.0)	51.0 (45.0–57.0)	0.089
Body mass index (kg/m^2^)	24.5 (22.7–26.0)	24.3 (22.6–26.3)	0.719
Waist circumference (cm)	86.0 (81.0–91.0)	86.0 (81.0–91.4)	0.722
Alcohol consumer	280 (72.5%)	579 (79.5%)	0.010
Current smoker	155 (40.2%)	298 (40.9%)	0.851
Routine exercise	99 (25.6%)	174 (23.9%)	0.568
Hypertension	96 (24.9%)	147 (20.2%)	0.085
Diabetes	33 (8.5%)	89 (12.2%)	0.077
Systolic blood pressure (mmHg)	121.0 (111.8–131.0)	121.0 (113.0–131.0)	0.808
Diastolic blood pressure (mmHg)	78.0 (71.0–86.0)	78.0 (72.0–86.0)	0.600
**Laboratory tests**			
Haemoglobin (g/dL)	15.4 (14.6–16.0)	15.5 (14.8–16.1)	0.211
Glucose (mmol/L)	5.44 (5.05–5.94)	5.38 (5.00–5.94)	0.342
Total cholesterol (mmol/L)	5.00 (4.45–5.57)	5.15 (4.53–5.72)	0.011
HDL-cholesterol (mmol/L)	1.24 (1.08–1.47)	1.25 (1.09–1.46)	0.494
Triglyceride (mmol/L)	1.50 (1.06–2.12)	1.43 (0.98–1.97)	0.068
Aspartate aminotransferase (U/L)	22.0 (18.0–27.0)	26.0 (21.0–33.0)	<0.001
Alanine aminotransferase (U/L)	25.0 (19.0–37.0)	30.0 (22.0–43.0)	<0.001
Blood urea nitrogen (mmol/L)	5.05 (4.32–5.89)	4.39 (3.71–5.28)	<0.001
Creatinine (mg/dL)	1.1 (1.0–1.2)	1.1 (1.0–1.2)	0.474
Estimated GFR (mL/min/1.73 m^2^)	71.4 (63.3–78.5)	70.3 (64.1–77.9)	0.749
**Sex hormone-related factors**			
Total testosterone (nmol/L)	15.72 (12.38–19.92)	14.89 (11.63–18.31)	<0.001
Free testosterone (nmol/L)	0.279 (0.224–0.341)	0.256 (0.203–0.323)	<0.001
Sex hormone-binding globulin (nmol/L)	40.67 (31.29–54.12)	39.93 (29.72–52.08)	0.449
**Electrolytes**			
Sodium (mEq/L)	141.5 (140.0–143.0)	143.0 (142.0–144.0)	<0.001
Potassium (mEq/L)	4.2 (4.1–4.4)	3.9 (3.7–4.1)	<0.001
Chloride (mEq/L)	103.0 (102.0–105.0)	104.0 (102.0–105.0)	0.049
Calcium (mmol/L)	2.35 (2.28–2.43)	2.25 (2.18–2.35)	<0.001
Phosphorus (mmol/L)	1.07 (1.00–1.20)	2.10 (1.91–2.33)	<0.001
Urinalysis*			
Urine ketone body (+)	26 (6.8%)	22 (3.0%)	0.006
Urine specific gravity (≥1.030)	111 (28.8%)	624 (86.1%)	<0.001

The values are presented as median (interquartile range) or number (proportion).

*Urine samples were not collected in 4 subjects.

HDL, high-density lipoprotein; GFR, glomerular filtration rate.

The correlation between the levels of TT and FT and the metabolic factors were analysed (Table 2). TT was positively correlated with SHBG and high-density lipoprotein (HDL)-cholesterol, and negatively correlated with waist circumference, glucose, and triglyceride in both groups. The correlation between TT and age was significantly positive in the bowel preparation group (r = 0.103). In the bowel preparation group, calcium and phosphorus were significantly correlated with FT, but not with TT.

**Table 2:** Pearson correlation coefficients of the variables for the levels of total and free testosterone.

	Total testosterone				Free testosterone			
	Bowel preparation (−)		Bowel preparation (+)		Bowel preparation (−)		Bowel preparation (+)	
	r	P	r	P	r	P	r	P
Age	−0.050	0.323	0.103	0.006	−0.327	<0.001	−0.187	<0.001
Systolic blood pressure	−0.044	0.384	−0.111	0.003	0.019	0.717	−0.109	0.003
Diastolic blood pressure	−0.007	0.895	−0.041	0.267	0.053	0.297	−0.018	0.626
SHBG (log)	0.485	<0.001	0.509	<0.001	−0.224	<0.001	−0.221	<0.001
Body mass index	−0.086	0.093	−0.170	<0.001	0.172	<0.001	0.050	0.178
Waist circumference	−0.152	0.003	−0.190	<0.001	0.048	0.345	0.029	0.436
Glucose (log)	−0.134	0.009	−0.078	0.035	−0.091	0.074	−0.036	0.336
Total cholesterol	0.033	0.513	−0.030	0.413	0.034	0.503	0.023	0.529
HDL-cholesterol	0.171	<0.001	0.124	<0.001	0.011	0.832	0.004	0.921
Triglyceride (log)	−0.125	0.014	−0.117	0.002	0.095	0.063	0.106	0.004
Aspartate aminotransferase	−0.045	0.381	0.041	0.272	−0.074	0.146	−0.039	0.292
Alanine aminotransferase	−0.097	0.056	−0.029	0.441	−0.052	0.304	0.017	0.641
Estimated GFR	−0.035	0.493	0.003	0.941	−0.020	0.701	−0.017	0.642
Calcium	0.062	0.223	0.060	0.108	0.042	0.412	0.088	0.017
Phosphorus	−0.005	0.929	−0.043	0.247	−0.002	0.971	−0.086	0.021

SHBG, sex hormone-binding globulin; HDL, high-density lipoprotein; GFR, glomerular filtration rate.

**Figure 1:**
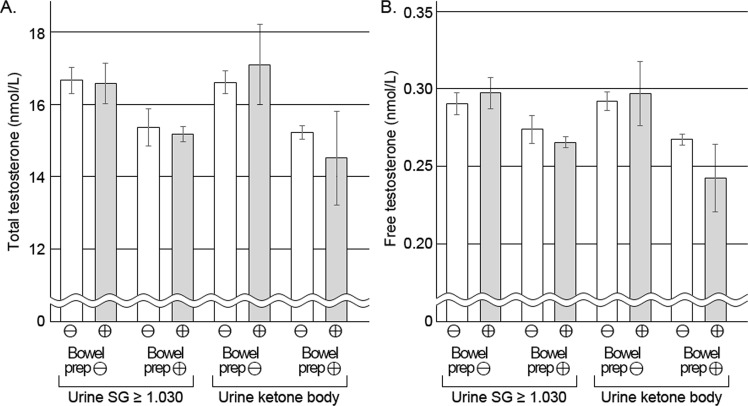
Mean levels of total and free testosterone according to dehydration. The levels of total (**A**) and free testosterone (**B**) were not significantly different according to dehydration defined by urine specific gravity or ketone body formation in both groups of bowel preparation and control. SG, specific gravity.

**Figure 2:**
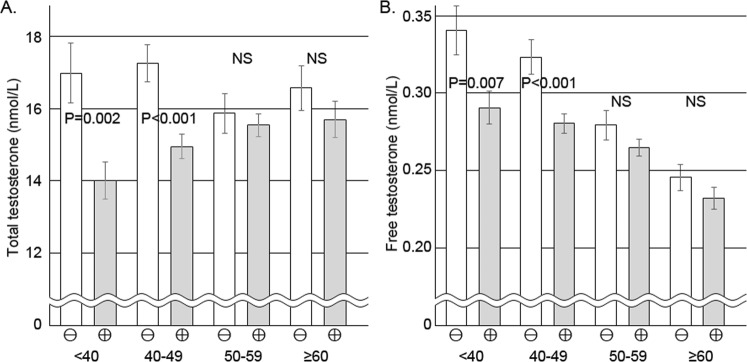
Mean levels of total and free testosterone according to age groups. Bowel preparation group had a significantly lower total (**A**) and free testosterone (**B**) than the control group in younger age groups (<40 and 40–49 years). However, no significant difference between bowel preparation groups and controls was seen in the older groups.

The levels of TT and FT were compared between men with a high urine specific gravity and the others, and between men with urine ketone body and those without ketone body (Fig. 1). The levels of TT and FT were not significantly different according to the criteria of urine specific gravity or urine ketone body. The levels of TT and FT were compared between the bowel preparation and control groups according to age groups (Fig. 2). In younger groups (aged <40 and 40–49), the levels of TT and FT were significantly different between the 2 groups. However, in older age groups (aged 50–59 and 60 or older), the differences were not significant between the 2 groups.

Multivariate regression models were formulated using candidate factors influencing the levels of TT and FT according to the bowel preparation and control groups, respectively (Table 3). In the model for TT, age was a significant factor in the control group, but not in the bowel preparation group. TT level was negatively correlated with the phosphorus level only in the bowel preparation group. Blood pressure (BP), HDL-cholesterol, and triglyceride were significantly associated with TT levels only in the bowel preparation group. BP, triglyceride, and phosphorus were significantly associated with FT in the bowel preparation group in FT models. The majority of variables, excepting age and smoking, that were included in the models for FT were not significant in the control groups.

**Table 3:** Unstandardized regression coefficients of the variables for the levels of total and free testosterone according to bowel preparation.

	Total testosterone				Free testosterone			
	Bowel preparation (−)		Bowel preparation (+)		Bowel preparation (−)		Bowel preparation (+)	
	B (SE)	P	B (SE)	P	B (SE)	P	B (SE)	P
Age (10 years)	−1.25 (0.30)	<0.001	−0.36 (0.23)	0.113	−0.03 (0.01)	<0.001	−0.01 (0.00)	0.001
SHBG (log in nmol/L)	8.05 (0.72)	<0.001	6.88 (0.47)	<0.001				
Systolic BP (10 mmHg)	0.25 (0.20)	0.221	−0.40 (0.13)	0.003	0.00 (0.00)	0.307	−0.01 (0.00)	0.002
Waist circumference (10 cm)	0.13 (0.36)	0.717	0.06 (0.24)	0.800	0.01 (0.01)	0.163	0.01 (0.00)	0.208
Glucose (log in mmol/L)	−1.14 (1.71)	0.505	−1.75 (1.06)	0.097	−0.03 (0.04)	0.375	−0.03 (0.02)	0.203
HDL-cholesterol (mmol/L)	0.56 (1.00)	0.578	1.29 (0.61)	0.035	0.01 (0.02)	0.787	0.02 (0.01)	0.072
Triglyceride (log in mmol/L)	0.19 (0.62)	0.758	1.00 (0.39)	0.009	0.01 (0.01)	0.695	0.02 (0.01)	0.004
Calcium (mmol/L)	0.92 (2.30)	0.689	1.98 (1.06)	0.062	0.00 (0.05)	0.935	0.03 (0.02)	0.106
Phosphorus (mmol/L)	−1.45 (1.48)	0.327	−1.09 (0.47)	0.021	−0.04 (0.03)	0.255	−0.02 (0.01)	0.038
Current smoker	1.12 (0.57)	0.048	0.69 (0.36)	0.055	0.03 (0.01)	0.028	0.01 (0.01)	0.154
Alcohol consumer	0.59 (0.61)	0.333	−0.08 (0.43)	0.848	0.01 (0.01)	0.372	0.00 (0.01)	0.795
Routine exercise	−0.70 (0.61)	0.251	−0.12 (0.40)	0.757	−0.01 (0.01)	0.421	0.00 (0.01)	0.892
Hypertension	−0.17 (0.65)	0.798	−0.28 (0.44)	0.525	0.00 (0.01)	0.894	−0.01 (0.01)	0.375
Diabetes	−0.60 (1.03)	0.558	0.86 (0.60)	0.148	0.00 (0.02)	0.970	0.02 (0.01)	0.148

SHBG, sex hormone-binding globulin; BP, blood pressure; HDL, high-density lipoprotein.

Multivariate regression models for TT and FT were formulated including age, bowel preparation, their interaction, and potential confounders (Table 4). The variables of age, bowel preparation, and their interaction terms were significant in both models for TT and FT. Smoking and triglyceride were also significantly related to TT and FT.

**Table 4:** Standardized regression coefficients of the variables for the levels of total and free testosterone.

	Total testosterone		Free testosterone	
	Beta (SE)	P	Beta (SE)	P
Age	−0.09 (0.04)	0.021	−0.14 (0.04)	<0.001
SHBG (log)	0.56 (0.03)	<0.001		
Systolic blood pressure	−0.04 (0.03)	0.131	−0.05 (0.03)	0.104
Waist circumference	0.01 (0.03)	0.673	0.06 (0.03)	0.071
Glucose (log)	−0.05 (0.03)	0.097	−0.05 (0.03)	0.142
HDL-cholesterol	0.06 (0.03)	0.037	0.05 (0.03)	0.112
Triglyceride (log)	0.07 (0.03)	0.012	0.09 (0.03)	0.007
Bowel preparation	−0.13	<0.001	−0.14	<0.001
Interaction between age and bowel preparation	0.08	0.033	0.11	0.012
Current smoker	0.07	0.009	0.07	0.021
Alcohol consumer	0.02	0.559	0.01	0.638
Routine exercise	−0.02	0.425	−0.02	0.563
Hypertension	−0.01	0.735	−0.01	0.654
Diabetes	0.02	0.490	0.03	0.353

SHBG, sex hormone-binding globulin; BP, blood pressure; HDL, high-density lipoprotein.

## Discussion

In the present study, the levels of serum TT and FT were significantly lower in subjects with bowel preparation than the ones in the control groups. The difference of TT and FT between the 2 groups was higher in younger men than in older men. The interaction between bowel preparation and age was a significant factor influencing serum levels of androgens.

Bowel preparation for colonoscopy may induce various adverse events. The most harmful adverse event caused by bowel preparation using sodium phosphate is phosphate nephropathy^30,31^. The effects are known to be related to age and dose^32,33^. Sodium phosphate can cause the imbalance of fluid and electrolyte in vulnerable subjects^34–36^. In our study, bowel preparation induced imbalances in various electrolytes and resulted especially in hyperphosphatemia. However, a significant difference in indices of renal function was not seen between the 2 groups. This suggests that sodium phosphate can induce electrolyte imbalances even though it does not cause significant nephropathy. On the other side, nephropathy is not a major cause of the androgen decrease shown in the bowel preparation group of our study. The electrolyte imbalance may be a candidate in causing androgen decline. However, although the hyperphosphatemia was more prevalent in the elderly as expected (data not shown), TT level was not correlated with phosphorus concentrations without adjusting for age in both groups. The sodium phosphate-induced hyperphosphatemia might be influenced by age and play only a partial role in reducing the circulating androgen. Some studies have reported that sodium phosphate may also cause elevated blood urea nitrogen levels, hypocalcaemia^18,37^, hyponatremia, and seizures^26,38^. However, the subjects undergoing bowel preparation in our study did not show these findings except for hypocalcaemia.

Common symptoms like diarrhoea and vomiting may induce dehydration during the process of bowel preparation^18^. In a previous study, the degree of dehydration was inversely related with TT levels^27,39^. However, although our study showed that bowel preparation might elevate urine specific gravity, dehydration assessed by urine specific gravity or ketone body was not related with levels of TT and FT in both bowel preparation and control groups.

Bowel preparation commonly causes anal irritation, abdominal cramps, abdominal distention, nausea as well as diarrhoea and vomiting^18,19^. These events easily cause pain and sleep disturbances^40^. Hence, bowel preparation can be considered an iatrogenic acute stress condition such as acute disease or surgery. Studies have reported the correlation between pain or sleep disturbances and testosterone^41–43^. Low levels of testosterone were reported in acute stress conditions^44^. According to previous studies, testosterone level was inversely correlated with cortisol level^45,46^. Cortisol is secreted in stress conditions, acting upon the HPG axis to induce negative feedback, and finally inhibiting the production of testosterone at gonad level^47,48^. The activation of the sympathetic tone may also cause vasoconstriction in the gonads, resulting in a decreased testosterone synthesis^12,49^.

The differences in androgen levels between the bowel preparation group and the control group were higher in younger subjects. Contrasting the control group, the levels of TT were significantly increasing by age in the bowel preparation group. These results implied that bowel preparation may reduce circulating testosterone, especially in younger men. Considering that hyperphosphatemia was more induced by bowel preparation in older men participating in the study, lower testosterone levels in younger men were difficult to predict. Moreover, the interaction between age and bowel preparation for TT and FT levels was significant in the multivariate regression models. It can be suggested that the influence of bowel preparation on the levels of testosterone depends on age. It is known that the response of the hypothalamic-pituitary-adrenal (HPA) and HPG axes to acute stress decreases with age^50–52^. It is also known that circadian changes of testosterone decrease in elderly. Therefore, it may be believed that the reduction in TT under the same stress may be higher in younger men than in older men. It may be inferred that bowel preparation represents a sufficient stress that influences the HPG axis, and that it is incredible to collect blood samples to measure testosterone serially after bowel preparation especially in young men.

Low levels of testosterone are recognized as a metabolic disorder^53–56^. Our study also showed that the level of TT is well correlated with metabolic parameters such as waist circumference, glucose, HDL-cholesterol, and triglyceride. However, most parameters did not have significance in the multivariate model including the variable of SHBG. Similarly, FT levels calculated using SHBG levels were not well correlated with most metabolic parameters in the univariate or multivariate models. These findings are consistent with those of the previous studies^57–62^. It may be inferred that SHBG is an important metabolic marker.

This study has some limitations. First, the current study was conducted in a cross-sectional manner. If TT levels data can be collected before and after bowel preparation, longitudinal analysis can be conducted. To reduce study design error, a large control sample was used in our analysis. Second, important hormonal factors consisting of players in the HPA and HPG axes were not evaluated. Further measurements of cortisol, luteinizing hormone, adrenocorticotropic hormone, et cetera may help in interpreting the results from a variety of viewpoints. Third, we did not survey the severity of common symptoms such as vomiting, diarrhoea, and abdominal cramps after bowel preparation. The additional symptom data might further support our objective dehydration and electrolyte imbalance data.

The current study is the first study to investigate testosterone levels related to bowel preparation, an essential step in colonoscopy. The FDA has issued a box warning against using sodium phosphate due to the potential risk of renal injury and electrolyte imbalance^63^. Our study added that the low levels of testosterone may be associated with sodium phosphate used in bowel preparation regardless of renal dysfunction or electrolyte imbalance. Further studies should be conducted to determine whether testosterone levels are influenced by using sodium phosphate or by the bowel preparation process itself, and whether the observed levels are temporary or not.

In conclusion, bowel preparation may independently decrease the levels of serum TT and FT. The reduction in serum testosterone levels is prominent in young men. We do not recommend measuring serum testosterone concentration right after the bowel preparation for colonoscopy. The mechanisms of the testosterone level decline induced by bowel preparation may be investigated in the further studies.

## Methods

### Study population

The participants in this cross-sectional study were male adults who visited the hospital for health check-ups from January 2008 to May 2010. A total of 1161 subjects had serum TT and SHBG concentration levels measured. Among them, we excluded participants who were receiving hormone therapy. We also excluded patients with an acute disease, liver disease such as chronic hepatitis and cirrhosis, chronic kidney disease, and a history of stroke, angina, myocardial infarction, or any type of cancer. A total of 1114 men were finally included in the analyses.

### Clinical and laboratory parameters

We collected information regarding medical history and lifestyle habits of participants using self-report questionnaires. Smoking history was used to categorize participants into current smokers or not (ex-smokers and non-smokers). Drinking history was categorized into current consumer and non-consumer. Exercise was classified into routine (moderate-to-strenuous intensity, 3 times a week or more frequent) and non-routine. Body mass index was defined as body weight (kg) divided by the square of height (m^2^). Waist circumference was measured to the nearest 0.1 cm midway between the lower rib margin and the iliac crest in a standing position. BP was measured after resting for 10 minutes in a sitting position using an automatic sphygmomanometer (TM-2655; A&D Co.,Tokyo, Japan).

Venous blood samples were drawn from the antecubital area between 7:30 a.m. and 10:30 a.m. after an overnight fast of 8 h. Serum samples were stored at 4 °C and analysed within a day of sampling. Serum analyses were performed on a daily basis. Glucose, creatinine, liver enzymes, electrolytes, and lipid profiles were measured using an automatic analyser (Roche/Hitachi Modular Analytics D2400 & P800 module; Roche, Tokyo, Japan). Glomerular filtration rate was estimated using the Modification of Diet in Renal Disease method^64^. Urine samples were simultaneously collected with blood samples. Urinary ketone and urinary specific gravity were measured using an automated strip reader. (Sysmex UC 3500; Sysmex, Kobe, Japan).

TT was measured by radioimmunoassay using Coat-a-Count® Total Testosterone kit (Siemens Healthcare Diagnostics Inc., Tarrytown, NY, USA) with inter- and intra- assay coefficient of variation of 5.9–6.7%. SHBG levels were measured by electrochemiluminescence immunoassay, Cobas 6000® analyzer (Roche, Mannheim, Germany) with inter- and intra- assay coefficient of variation of 1.3% and 2.4%. FT levels were calculated using the Vermeulen’s method^65^.

### Bowel preparation

Bowel preparation before colonoscopy included the oral administration of 2 bottles of sodium phosphate. All participants received a 90 mL sodium phosphate solution (Phosphanol®, DONGINDANG Pharmaceutical Company, Gyeonggi-do, Korea: each 100 mL containing 48 g sodium dihydrogen phosphate and 18 g sodium hydrogen phosphate) for bowel preparation. At 7 p.m. the day before, half of the sodium phosphate solution (45 mL) was mixed with a cup of water (240 mL) and at least 3 cups of water were consumed at 10:00 p.m. At 5:00 a.m. on the day of the test, the remaining half of the sodium phosphate solution (45 mL) was mixed with a cup of water (240 mL) and then 2–3 additional cups of water were consumed before the health examination.

### Statistical analyses

For the descriptive analysis, the results were expressed as median (interquartile range) or number (proportion). The difference in variables between the bowel preparation and control groups were analysed using Mann-Whitney U test or chi-square test. Skewed variables were used in the subsequent analyses after being logarithmically transformed. Pearson correlation analyses were used to investigate the linear relationship of the levels of TT and FT with age; the levels of SHBG, liver enzymes, calcium, and phosphorus; renal function; and metabolic parameters. To compare the levels of TT and FT according to dehydration and age groups, independent sample t-test and one-way ANOVA were used for the bowel preparation and control groups, respectively. To find the factors influencing the levels of TT and FT, multivariate regression models were constructed based on the results of the correlation analyses according to the 2 groups, respectively. Finally, to confirm the influence of bowel preparation, age, and their interaction on the levels of TT and FT, potential factors were included in a single regression model.

All analyses were conducted using SPSS version 21.0 statistical analysis software (IBM, Armonk, NY, USA). P < 0.05 was considered statistically significant.

### Ethics approval and consents to participate

All participants were unconditioned volunteers in our study. Informed consent was obtained from all participants, and the study was approved by the Institutional Review Board of the CHA Bundang Medical Center, Korea (BD2013-081). All methods were performed in accordance with the relevant guidelines and regulations.

## References

[1] Mooradian AD, Morley JE, Korenman SG (1987). Biological actions of androgens. Endocr Rev.

[2] Wilson JD (1999). The role of androgens in male gender role behavior. Endocr Rev.

[3] Baskin HJ (2002). American Association of Clinical Endocrinologists medical guidelines for clinical practice for the evaluation and treatment of hyperthyroidism and hypothyroidism. Endocr Pract.

[4] Lejeune H, Huyghe E, Droupy S (2013). Hypoactive sexual desire and testosterone deficiency in men. Prog Urol.

[5] Lunenfeld B, Arver S, Moncada I, Rees DA, Schulte HM (2012). How to help the aging male? Current approaches to hypogonadism in primary care. Aging Male.

[6] Lunenfeld B (2015). Recommendations on the diagnosis, treatment and monitoring of hypogonadism in men. Aging Male.

[7] Bhasin S (2018). Testosterone Therapy in Men With Hypogonadism: An Endocrine Society Clinical Practice Guideline. J Clin Endocrinol Metab.

[8] Fisher CL, Mannino DM, Herman WH, Frumkin H (1997). Cigarette smoking and thyroid hormone levels in males. Int J Epidemiol.

[9] La Grange L, Jones TD, Erb L, Reyes E (1995). Alcohol consumption: biochemical and personality correlates in a college student population. Addict Behav.

[10] Woolf PD, Hamill RW, McDonald JV, Lee LA, Kelly M (1985). Transient hypogonadotropic hypogonadism caused by critical illness. J Clin Endocrinol Metab.

[11] Hardy MP (2005). Stress hormone and male reproductive function. Cell Tissue Res.

[12] Kamiya H (2003). Effect of simulated microgravity on testosterone and sperm motility in mice. J Androl.

[13] Rabin D, Gold PW, Margioris AN, Chrousos GP (1988). Stress and reproduction: physiologic and pathophysiologic interactions between the stress and reproductive axes. Adv Exp Med Biol.

[14] Collu R, Gibb W, Ducharme JR (1984). Effects of stress on the gonadal function. J Endocrinol Invest.

[15] Kamel F, Kubajak CL (1987). Modulation of gonadotropin secretion by corticosterone: interaction with gonadal steroids and mechanism of action. Endocrinology.

[16] Rex DK, Imperiale TF, Latinovich DR, Bratcher LL (2002). Impact of bowel preparation on efficiency and cost of colonoscopy. Am J Gastroenterol.

[17] Lee BI (2012). Korean guidelines for colorectal cancer screening and polyp detection. Clin Endosc.

[18] Holte K, Nielsen KG, Madsen JL, Kehlet H (2004). Physiologic effects of bowel preparation. Dis Colon Rectum.

[19] Curran MP, Plosker GL (2004). Oral sodium phosphate solution: a review of its use as a colorectal cleanser. Drugs.

[20] Rabeneck L (2008). Bleeding and perforation after outpatient colonoscopy and their risk factors in usual clinical practice. Gastroenterology.

[21] Mitchell RM, McCallion K, Gardiner KR, Watson RG, Collins JS (2002). Successful colonoscopy; completion rates and reasons for incompletion. Ulster Med J.

[22] Shah HA, Paszat LF, Saskin R, Stukel TA, Rabeneck L (2007). Factors associated with incomplete colonoscopy: a population-based study. Gastroenterology.

[23] Lee KJ (2015). Electrolyte changes after bowel preparation for colonoscopy: A randomized controlled multicenter trial. World J Gastroenterol.

[24] Toledo TK, DiPalma JA (2001). Review article: colon cleansing preparation for gastrointestinal procedures. Aliment Pharmacol Ther.

[25] Committee ASoP (2015). Bowel preparation before colonoscopy. Gastrointest Endosc.

[26] Florentin M, Liamis G, Elisaf MS (2014). Colonoscopy preparation-induced disorders in renal function and electrolytes. World J Gastrointest Pharmacol Ther.

[27] Judelson DA (2008). Effect of hydration state on resistance exercise-induced endocrine markers of anabolism, catabolism, and metabolism. J Appl Physiol.

[28] Maresh CM (2006). Effect of hydration state on testosterone and cortisol responses to training-intensity exercise in collegiate runners. Int J Sports Med.

[29] Sanchez-Capelo A, Castells MT, Cremades A, Penafiel R (1996). Hypokalemia decreases testosterone production in male mice by altering luteinizing hormone secretion. Endocrinology.

[30] Gonlusen G, Akgun H, Ertan A, Olivero J, Truong LD (2006). Renal failure and nephrocalcinosis associated with oral sodium phosphate bowel cleansing: clinical patterns and renal biopsy findings. Arch Pathol Lab Med.

[31] DiPalma JA, Buckley SE, Warner BA, Culpepper RM (1996). Biochemical effects of oral sodium phosphate. Dig Dis Sci.

[32] Rex DK (2007). Dosing considerations in the use of sodium phosphate bowel preparations for colonoscopy. Ann Pharmacother.

[33] Rostom A (2006). A randomized prospective trial comparing different regimens of oral sodium phosphate and polyethylene glycol-based lavage solution in the preparation of patients for colonoscopy. Gastrointest Endosc.

[34] Casais MN (2009). Hyperphosphatemia after sodium phosphate laxatives in low risk patients: prospective study. World J Gastroenterol.

[35] Khurana A, McLean L, Atkinson S, Foulks CJ (2008). The effect of oral sodium phosphate drug products on renal function in adults undergoing bowel endoscopy. Arch Intern Med.

[36] Afridi SA, Barthel JS, King PD, Pineda JJ, Marshall JB (1995). Prospective, randomized trial comparing a new sodium phosphate-bisacodyl regimen with conventional PEG-ES lavage for outpatient colonoscopy preparation. Gastrointest Endosc.

[37] Clarkston WK (1996). Oral sodium phosphate versus sulfate-free polyethylene glycol electrolyte lavage solution in outpatient preparation for colonoscopy: a prospective comparison. Gastrointest Endosc.

[38] Frizelle FA, Colls BM (2005). Hyponatremia and seizures after bowel preparation: report of three cases. Dis Colon Rectum.

[39] Irfan Y (2015). Associations among dehydration, testosterone and stress hormones in terms of body weight loss before competition. Am J Med Sci.

[40] Sharara AI (2016). The burden of bowel preparations in patients undergoing elective colonoscopy. United European Gastroenterol J.

[41] Opstad PK (1992). The hypothalamo-pituitary regulation of androgen secretion in young men after prolonged physical stress combined with energy and sleep deprivation. Acta Endocrinol (Copenh).

[42] Jauch-Chara K, Schmid SM, Hallschmid M, Oltmanns KM, Schultes B (2013). Pituitary-gonadal and pituitary-thyroid axis hormone concentrations before and during a hypoglycemic clamp after sleep deprivation in healthy men. PLoS One.

[43] Schmid SM, Hallschmid M, Jauch-Chara K, Lehnert H, Schultes B (2012). Sleep timing may modulate the effect of sleep loss on testosterone. Clin Endocrinol (Oxf).

[44] Aono T, Kurachi K, Miyata M, Nakasima A, Koshiyama K (1976). Influence of surgical stress under general anesthesia on serum gonadotropin levels in male and female patients. J Clin Endocrinol Metab.

[45] Brownlee KK, Moore AW (2005). & Hackney, A. C. Relationship between circulating cortisol and testosterone: influence of physical exercise. J Sports Sci Med.

[46] Daly W, Seegers CA, Rubin DA, Dobridge JD, Hackney AC (2005). Relationship between stress hormones and testosterone with prolonged endurance exercise. Eur J Appl Physiol.

[47] Chen S, Wang J, Yu G, Liu W, Pearce D (1997). Androgen and glucocorticoid receptor heterodimer formation. A possible mechanism for mutual inhibition of transcriptional activity. J Biol Chem.

[48] Viau V (2002). Functional cross-talk between the hypothalamic-pituitary-gonadal and -adrenal axes. J Neuroendocrinol.

[49] Kraut A, Barbiro-Michaely E, Mayevsky A (2004). Differential effects of norepinephrine on brain and other less vital organs detected by a multisite multiparametric monitoring system. Med Sci Monit.

[50] Goya RG, Bolognani F, Herenu CB, Rimoldi OJ (2001). Neuroendocrinology of aging: the potential of gene therapy as an interventive strategy. Gerontology.

[51] Liu PY, Iranmanesh A, Nehra AX, Keenan DM, Veldhuis JD (2005). Mechanisms of hypoandrogenemia in healthy aging men. Endocrinol Metab Clin North Am.

[52] Veldhuis JD, Iranmanesh A (2005). Short-term aromatase-enzyme blockade unmasks impaired feedback adaptations in luteinizing hormone and testosterone secretion in older men. J Clin Endocrinol Metab.

[53] Kapoor D, Malkin CJ, Channer KS, Jones TH (2005). Androgens, insulin resistance and vascular disease in men. Clin Endocrinol (Oxf).

[54] De Pergola G (2000). The adipose tissue metabolism: role of testosterone and dehydroepiandrosterone. Int J Obes Relat Metab Disord.

[55] Yanase T (2008). Androgens and metabolic syndrome: lessons from androgen receptor knock out (ARKO) mice. J Steroid Biochem Mol Biol.

[56] Pitteloud N (2005). Relationship between testosterone levels, insulin sensitivity, and mitochondrial function in men. Diabetes Care.

[57] Tsai EC, Matsumoto AM, Fujimoto WY, Boyko EJ (2004). Association of bioavailable, free, and total testosterone with insulin resistance: influence of sex hormone-binding globulin and body fat. Diabetes Care.

[58] Garaulet M, Perex-Llamas F, Fuente T, Zamora S, Tebar FJ (2000). Anthropometric, computed tomography and fat cell data in an obese population: relationship with insulin, leptin, tumor necrosis factor-alpha, sex hormone-binding globulin and sex hormones. Eur J Endocrinol.

[59] Makinen JI (2008). Endogenous testosterone and serum lipids in middle-aged men. Atherosclerosis.

[60] Agledahl I, Skjaerpe PA, Hansen JB, Svartberg J (2008). Low serum testosterone in men is inversely associated with non-fasting serum triglycerides: the Tromso study. Nutr Metab Cardiovasc Dis.

[61] Akishita M (2010). Association of low testosterone with metabolic syndrome and its components in middle-aged Japanese men. Hypertens Res.

[62] Hong D (2013). Total testosterone and sex hormone-binding globulin are associated with metabolic syndrome independent of age and body mass index in Korean men. Maturitas.

[63] U.S. Food and Drug Administration. Oral Sodium Phosphate (OSP) Products for Bowel Cleansing (marketed as Visicol and OsmoPrep, and oral sodium phosphate products available without a prescription). Available at, http://www.fda.gov/drugs/drugsafety/postmarketdrugsafetyinformationforpatientsandproviders/ucm103354.htm. Accessed March 8 (2014).

[64] Levey AS (2009). A new equation to estimate glomerular filtration rate. Ann Intern Med.

[65] Vermeulen A, Verdonck L, Kaufman JM (1999). A critical evaluation of simple methods for the estimation of free testosterone in serum. J Clin Endocrinol Metab.

